# Mechanism of blood-retinal barrier breakdown induced by HIV-1 (Review)

**DOI:** 10.3892/etm.2014.1521

**Published:** 2014-02-06

**Authors:** XIN CHE, XIAN-QUN FAN, ZHI-LIANG WANG

**Affiliations:** 1 Department of Ophthalmology, Ninth People’s Hospital Affiliated with Shanghai Jiaotong University, Shanghai 200011, P.R. China; 2 Key Laboratory of Ophthamology, Ninth People’s Hospital Affiliated with Shanghai Jiaotong University, Shanghai 200011, P.R. China

**Keywords:** actin cytoskeleton, blood-retinal barrer, caveolin-1, matrix metalloproteinases, ocular HIV-1, tight junction

## Abstract

Human immunodeficiency virus (HIV)-1 has been detected in ocular tissues; however, the mechanism of entry has not been established. It has been hypothesized that the blood-retinal barrier (BRB), a critical guardian against microbial invasion of the eye, may be compromised in the presence of HIV-1 in the eye. *In vivo* and *in vitro* model systems have shown that the breach of tight junctions induced by HIV-1-associated factors contributes to the breakdown of the BRB. The present study reviews the mechanism of tight junction disruption, focusing on signaling pathways, the expression of enzymes, including metalloproteinases, and cytokines that affect inflammation. The studied pathways may be potential targets for the prevention of ocular HIV complications.

## 1. Introduction

Han *et al* performed a cross-sectional study on a group of human immunodeficiency virus (HIV)-1-infected patients who underwent long-term highly active antiretroviral therapy. The results indicated that various fragments of the HIV-1 genome were detectable in tears, in the absence of a detectable plasma viral load (1). Earlier in the 1980s, studies isolated HIV viruses from tears, cornea, aqueous humor, conjunctiva, retinal vascular endothelium and even contact lenses (2–4). Pathanapitoon *et al* analyzed the aqueous and vitreous humor samples from HIV-1-infected patients and observed that several patients had intraocular HIV-1 RNA levels that were higher than the corresponding HIV-1 RNA plasma levels, which indicated a largely elevated ocular-to-plasma HIV ratio (5). Thus, the mechanisms by which HIV invades the eye and exists in the tissues in the absence of a detectable plasma virus level were questioned. To date, there has been no explanation of these circumstances. A growing number of studies have shown that the central nervous system (CNS) is a sanctuary for HIV, which crosses the blood-brain barrier (BBB) early in the course of systemic infection and resides in brain macrophages and microglia (6,7). One hypothesis is that HIV persists in these sanctuaries during antiretroviral treatment and may cause the generation and dissemination of drug-resistant viruses (8). Another hypothesis is that the breakdown of the blood-retinal barrier (BRB), which is associated with the changes in the tight junctions, contributes to the trafficking of HIV into the eye (9,10). Therefore, the present review focused on the key breakdown mechanisms of tight junctions.

## 2. Components of the blood-retinal barrier

**Figure 1:**
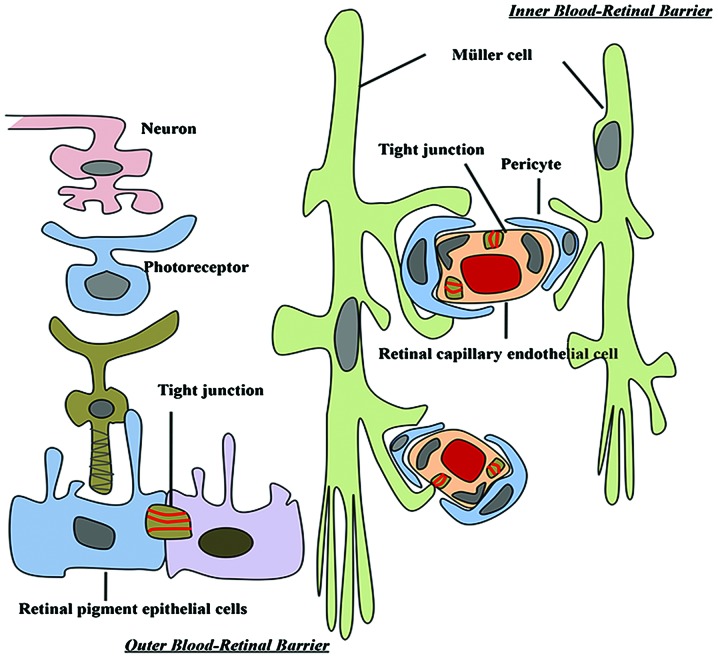
Retinal-vascular unit and tight junctions between endothelial cells, forming the inner and outer blood-retinal barrier.

The BBB provides significant protection against microbial invasion of the brain (11). The BRB and BBB are derived from the same embryonic primordium. Brain endothelial cells form extremely tight cell-cell junctions that are distinct from the tight junctions of endothelia and epithelia elsewhere in the body. Brain endothelial cells lack fenestrations and have a high number of mitochondria, which are characteristics associated with their specialized functions. For example, a high mitochondrial content is likely to be important for providing the energy required to maintain the structure and function of the BBB (12). For BBB capillaries, the transendothelial electrical resistance, an indicator of permeability, ranges between 1,000 and 2,000 Ω/cm^2^. However, for systemic capillaries this value is only 5–10 Ω/cm^2^. The BRB, which maintains eye homeostasis, has a similar nature to the BBB (13). The BRB is composed of retinal capillary endothelial cells (inner BRB) and retinal pigment epithelium (RPE) cells (outer BRB) (14). These two cell types develop tight junctions that confer a high degree of control of solute and fluid permeability between the circulating blood and the neural retina (Fig. 1).

## 3. Tight junctions in the eye

**Figure 2:**
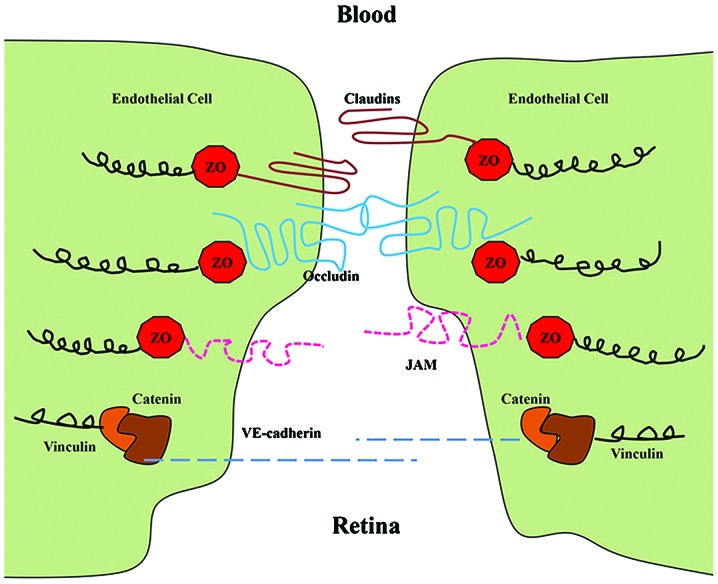
Major molecules of the tight and adherens junctions are shown. Tight junction proteins include ZO, occludin, claudins and JAMs, while adherens junction proteins include catenins and vinculins. JAMs, junctional adhesion molecules; ZO, zonula occludens.

The transmembrane proteins of tight junctions include occludin, junction adhesion molecules and claudins. These proteins extend into the paracellular space, acting in concert to affect barrier properties (15). Occludin and claudins have external loops that mediate intercellular adhesion by interaction with occludin and claudins of neighboring cells (16). In addition, claudins and occludin interact with zonula occludens (ZOs) −1, −2 and −3, which in turn associate with the actin cytoskeleton (Fig. 2). The 220-kDa phosphoprotein ZO-1, in particular, is able to bind to a wide variety of protein partners and allow for the control of tight junction assembly (17). During viral infections and other pathological conditions, altering the localization or cleavage of the tight junction proteins is the main pathological change, which results in the increasing permeability of the barrier (18).

## 4. Claudins

Claudin-5 is expressed predominantly in endothelial cells (19). A study using claudin-5-deficient mice demonstrated that it is necessary to preserve the vascular barrier to small (<0.8 kDa) molecules in the brain (20). As claudin-5 is expressed in the retinal vasculature (21), it is likely to contribute to the function of the BRB. The expression of claudin-3, −10 and −19 has been detected in the human fetal RPE (22).

## 5. Occludin

Increased expression of occludin has been observed to correlate with increased barrier function and decreased paracellular permeability (23,24). In addition, changes in the content and localization of occludin in diabetic retinas have been demonstrated to be associated with alterations in barrier function (25). A study of bovine retinal endothelial cells treated with vascular endothelial growth factor (VEGF) revealed a decreased occludin content and immunostaining at cell borders concomitant with increased BRB permeability (26). Similarly, diabetes reduces occludin content in the brain vasculature; this reduction correlates with the incidence of vascular diseases (27).

## 6. Zonula occludens-1

ZO proteins are intracellular proteins that associate with the cytoplasmic surface of tight junctions and organize the tight junction complex. The presence of ZO-1 is readily observed in retinal vascular endothelial and RPE cells. In these cell types, agents that induce permeability, including VEGF or hepatocyte growth factor, induce the redistribution of ZO-1 from the cell border to the cell interior (28,29).

## 7. Mechanism for the disruption of tight junctions

Increased permeability of the BRB may occur through two pathways, the paracellular or the transcellular pathway. The paracellular route is governed by tight junctions and is usually the main route of increased endothelial barrier permeability (30).

## 8. Tat-induced caveolae-associated signaling

Tat is the only protein actively secreted by HIV-1 infected cells. It circulates in the blood at high levels during HIV infection and crosses the BBB with large quantities entering the CNS (31). The VEGF receptor has been hypothesized to serve as a high-affinity receptor for Tat in endothelial cells (32). Tat specifically interacts with VEGF and surface molecules that belong to the large family of G-protein-coupled receptors localized to caveolae, to activate several protein kinases, including certain kinases involved in Ras signaling (33). Ras proteins are small GTPases that cycle between inactive GDP-bound and active GTP-bound conformations. Several elements of the Ras signaling cascades are localized in caveolae, the dominant type of lipid rafts in endothelial cells (34). Zhong *et al* focused on the breakdown mechanism of the BBB and found that Tat diminished the expression of several tight junction proteins, including occludin, ZO-1 and ZO-2, in the caveolar fraction of human brain microvascular endothelial cells (HBMECs) (35). These effects were effectively protected against by the pharmacological inhibition of Ras signaling and by the silencing of caveolin-1. Lin *et al* demonstrated that HIV infection in primary human monocyte-derived macrophages results in a marked upregulation of caveolin-1 expression mediated by the HIV Tat protein (36). Nag *et al* assessed the sequential expression of caveolae and occludin over a period of 12 h to 6 days post-lesion in a rat cortical cold injury model. The study demonstrated a significant increase in endothelial caveolin-1 expression in arterioles and large veins, particularly those with BBB breakdown to proteins (37). In addition, the HIV-1 Tat protein causes the paracellular permeability of RPE cells to increase *in vitro,* concomitant with changes in the expression of tight junctions. Therefore, the effects of Tat on the outer BRB may be mediated by Ras/ERK1/2 pathways (9).

## 9. Disruption of tight junctions and the basal lamina by secreted matrix metalloproteinases

The basal lamina of the BBB contains extracellular matrix molecules, including laminin, type IV collagen and fibronectin. The majority of these molecules are substrates for a family of neutral proteases called matrix metalloproteinases (MMPs), in particular MMP-2 and −9 (38). MMPs contribute to interactions between cells and the matrix, allowing movement and shape changes in CNS development and neuronal plasticity. MMPs are key mediators of tight junction protein alterations, which lead to BBB dysfunction (39,40). These zinc-dependent enzymes have proteolytic activity that acts on the extracellular matrix, including the basal laminae. MMPs are associated with tight junction disruption not only by basement membrane degradation, but also by cleavage of tight junction proteins (41,42). Elevated levels of MMP-9 have been reported in the cerebrospinal fluid of HIV-1-infected children (43) and adult patients (44). The overexpression of MMP-2 and MMP-9 was also reported in the brain of a severe combined immunodeficiency mouse model of HIV-1 encephalitis (45). In a study in rats, within 30 min of HIV-1 glycoprotein 120 (gp120) injection into the caudate-putamen (CP), MMP-2 co-localized with laminin and by 6 h there was a significant reduction in the number of laminin-positive structures in the injected CP. Similarly, the levels of vascular tight junction proteins, claudin-5 and occludin, were significantly decreased in the experimental group compared with those in the controls (46). In one study, primary HBMECs were exposed to HIV-1 Tat proteins. Tat induced MMP-9 expression, and RNA interference targeting MMP-9 reduced the paracellular permeability of Tat-treated HBMECs and the concentration of soluble occludin in the cell supernatant (47). In a diabetic rat model, the transepithelial electrical resistance (TER) was measured in the retinal endothelium and RPE following treatment with MMPs. The two cell types showed decreased TER and degradation of the tight junction proteins, indicating that elevated expression levels of MMPs in the retina may facilitate the change in BRB permeability (48).

## 10. Disruption of tight junctions by alterations in the actin cytoskeleton

Tight junctions may also be disrupted from within cells. Changes in the actin cytoskeleton are likely to occur upon alteration of the tight junction proteins, resulting in paracellular permeability changes (49). Reactive oxygen species play a role in disrupting tight junctions from within cells via the induction of the RhoA small GTPase, phosphoinositide 3-kinase and protein kinase B signaling pathways, concomitant with the rearrangement of the actin cytoskeleton and altered localization of occludin and claudin-5 (50). In addition, the alteration of the actin cytoskeleton induced by hypoxic stress correlates with changes in BBB permeability and ZO-1 localization (51). Bruban *et al* showed that disorganization of cytoskeletal actin filament models was accompanied by decreased expression of tight junction proteins by the RPE (52).

## 11. Inflammatory cytokines induce the destruction of tight junctions

A number of cytokines have been reported to be upregulated in the plasma of HIV-infected individuals or in plasma treated *ex vivo. In vitro*, the interaction of HIV or the HIV gp120 envelope with CD4 molecules induces the secretion of tumor necrosis factor (TNF)-α, interleukin (IL)-1 and other cytokines (53). Several studies have noted a marked increase in membrane permeability following exposure to vasoactive cytokines, including TNF-α, IL-1β, interferon-γ, histamine 65 and growth factors (54). When activated, inflammatory cells initiate the cellular release of free radicals, cytokines and growth factors (55). HIV-1 Tat is a strong proinflammatory agent that recruits and induces the transendothelial migration of monocytes (56). When Tat was injected into the hippocampi of mice, reductions in the levels of ZO-1 and its continuity were observed, in addition to inflammatory cell accumulation in the choroid plexus (57). It has also been shown that the expression of the occludin promoter is affected by TNF-α or interferon treatment (58). These studies indicate that the expression of HIV genes or proteins may alter the capacity of the cells to secrete important cytokines. Therefore, cytokines are likely to play a vital role in the pathology of HIV-associated complications.

## 12. Concluding remarks

The interplay between HIV-1 and hosts at the BRB is complex. The present review has shown that specific viral genes affect signaling pathways, the expression of enzymes, including MMPs, and cytokines that affect inflammation, which leads to the disruption of tight junctions. These effects are directly caused by HIV-1-associated proteins or HIV-1-induced inflammatory factors. This accumulating damage results in BRB breakdown. Advances in the understanding of HIV-host interactions are likely to be forthcoming as researchers apply effective approaches to their studies of this challenging topic. The present study reveals insights into the molecular mechanisms underlying BRB regulation and may provide opportunities for the treatment of ocular complications.
